# Detecting LNAPL migration in real time using electrical resistivity and statistical models

**DOI:** 10.1038/s41598-025-00702-2

**Published:** 2025-05-20

**Authors:** Ola Tareq al-Hussain, Harris Ramli, Mohammed J. Al-Haidarey, Hayder Yasir Naser

**Affiliations:** 1 School of Civil Engineering, Engineering Campus, Universiti Sains Malaysia, 14300 Nibong Tebal, Pulau Pinang Malaysia; 2 Environment and Pollution Engineering Department, Basrah Engineering Technical College, Southern Technical University, Basrah, Iraq; 3 Department of Ecology, Faculty of Science, University of Kufa, Al-Najaf, Iraq; 4 Department of Electrical Engineering, University of Basrah, Basrah, Iraq

**Keywords:** Soil contamination, Electrical resistivity, LNAPL assessment, Non-linear regression, Soil box design, Environmental sciences, Solid Earth sciences, Engineering, Materials science

## Abstract

To address the issue of detecting immediate Light Non-Aqueous Phase Liquid (LNAPL) spillage in subsurface soils, a refined method of Electrical Resistivity (ER) measurement was developed and evaluated in this study. A controlled experiment was conducted using a custom-designed nine-sector soil box to simulate stratified soil conditions. Diesel, an LNAPL surrogate, was injected at varying flow rates (5 ml/min, 25 ml/min, and 50 ml/min) to replicate immediate spillage scenarios. Electrical resistivity measurements were taken over 24 h using a multifunction installation tester, soil samples were analyzed to determine LNAPL concentration. The experimental results showed a significant decrease in ER values with increasing LNAPL concentration, particularly in loam and sandy loam soils, which exhibited higher permeability and facilitated faster LNAPL migration. Non-linear regression models—logarithmic, quadratic, and power—were applied to analyze the relationship between ER and LNAPL concentration. The power model demonstrated the best fit, showing a strong negative correlation (R² up to 0.942) and high statistical significance (*p* < 0.001). This study highlights the efficacy of ER as a real-time monitoring tool for detecting immediate LNAPL spillage and provides valuable insights into the influence of soil properties on LNAPL migration dynamics. These findings contribute to advancing geotechnical engineering practices and offer a foundation for developing rapid response strategies for environmental monitoring and remediation. Future research should expand on these findings by incorporating larger datasets and diverse soil conditions to further validate the observed relationships.

## Introduction

Large amounts of oil products seep into the ground, moving silently through soil and water and threatening nature and our health. Among these pollutants are Light Non-Aqueous Phase Liquids (LNAPLs), like gasoline, diesel, and jet fuel. Their significance lies within their extensive industrial use and a high potential for spillage^1^.

These substances pose serious risks when released into the environment as they can quickly infiltrate soil and groundwater, leading to long-lasting contamination that is difficult to remediate^2^ These compounds don’t mix with water and have lower densities, which is why they form a thin layer over the groundwater’s surface^3^.

LNAPL compounds are released into the environment in different mechanisms, such as accidental leakage from above-ground and underground storage tanks (USTs) with their associated pipelines, as well as unintentional release during handling, storage, or transfer at fuel manufacturing facilities, refineries, petrol filling stations^4,5^ ,

The main soil parameters that affect LNAPL are the Density, Viscosity, Capillary Pressure, Relative Permeability, Saturation, and hydraulic conductivity of a porous medium. These parameters greatly influence how LNAPL moves and behaves underground when it spills^6,7^.

Building on the spillage classification^8^, which noted that research has primarily focused on the three spillage types—Recent LNAPL Spillage, Aged Sites, and Legacy Sites—rather than Immediate Spillage^9^, and considering that LNAPL detection methods have varied, covering a range from basic sampling to complicated geophysical measurements as shown in the literature^10^, there is a need for better ways to detect and respond to immediate spill incidents. To address this need, Electrical Resistivity (ER) was used in this study as a method typically applied to understand underground conditions and detect pollutants like LNAPLs^11,12^.

Electrical Resistivity (ER) has been a key technique since being developed by^13^ to measure how strongly a material resists the flow of electrical current through it. In simpler terms, it tells us how easily or how difficulty electricity can pass through a substance^14–20^.

While efforts are being made to effectively identify LNAPL locations and accurately map contamination in real-time^21^, current literature reveals mixed results regarding the impact of LNAPL on electrical resistivity in soils. Studies such as those by^11,22^ suggest that extensively pre-testing soil samples before analyzing LNAPL movement could change the soil’s physical and chemical properties and heterogeneity, possibly leading to inaccurate data. Moreover, adjustments to the position of rods used in resistivity testing might remix the polluted soils, further altering their structure and skewing results. This variation in results was clear because some researchers, like^14,23^, have documented an increase in electrical resistivity with higher LNAPL concentrations, while others, including ^24^ and ^25^, have noted a decrease under similar conditions. Additionally, studies by^26^ have reported fluctuations in electrical resistivity without establishing a definitive trend related to LNAPL presence.

This inconsistency highlights the need for a more controlled and systematic approach to investigating the relationship between LNAPL concentration and ER values. Addressing this gap, the present study proposes a structured method to monitor LNAPL behavior under controlled conditions.

This study primarily aims to detect immediate LNAPL spillage using an advanced non-invasive electrical method and, secondly, to find a statistical relationship between ER values and LNAPL concentration in subsurface soil by constructing a nine-sector division soil box to monitor and assess the behavior of LNAPL movement. The measuring device was adapted for use in this specific soil-box setup. The novelty of this study is the combination of temporal soil-box experiments and statistical modeling.

## Methods

### Study site and data collection method

**Fig. 1:**
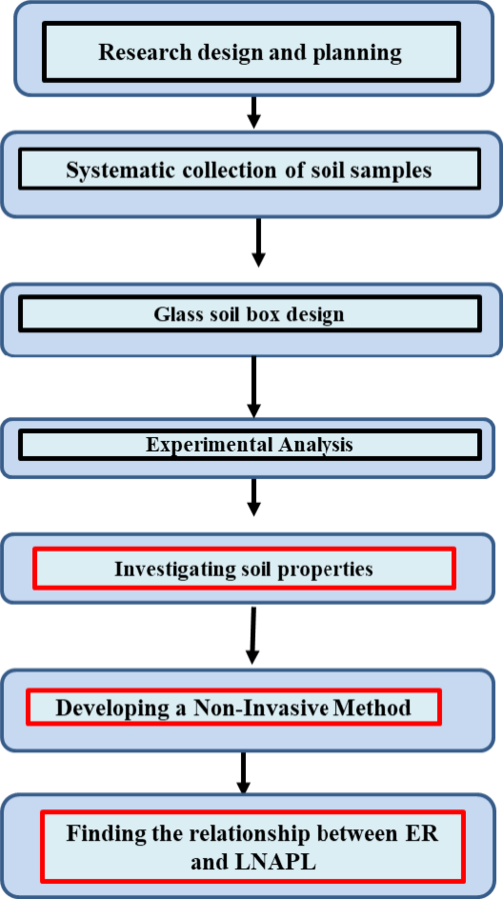
Overview of the research methodology.

The research was conducted in Bahar Al-Najaf, a region of ecological significance located in the southern part of Iraq, within the Middle Euphrates area, west of the Euphrates River. The samples were collected from a specific location at coordinates (31.9830520° latitude, 44.1712973° longitude), providing an ideal setting for investigating LNAPL migration and assessing soil dynamics.To provide a clear understanding of the overall structure of the research, a flowchart has been included to illustrate the key stages of the study in Fig. 1. The process begins with research design and soil sampling, then moves through the design of the experimental soil box and the analysis of soil properties. The final stages involve developing a non-invasive detection method and examining the relationship between electrical resistivity and LNAPL behavior.

### Soil sampling

The soil sampling process involved Systematic collection from various depths, ensuring the representation of the entire soil profile. Upon collection, the soil samples were divided into two distinct groups (group 1 and group 2). Group 1 underwent standard laboratory tests to assess its physical and chemical properties.

The other group (group 2) was delicately packed into the soil box to maintain the integrity of its natural structure for subsequent investigations^27–29^. Soil samples were transported in dark, airtight containers to prevent any chemical interactions or photo-oxidation, then cleaned from debris and checked for any metal pieces to prevent inaccurate readings of ER^30,31^ .

### Soil box setup

**Fig. 2:**
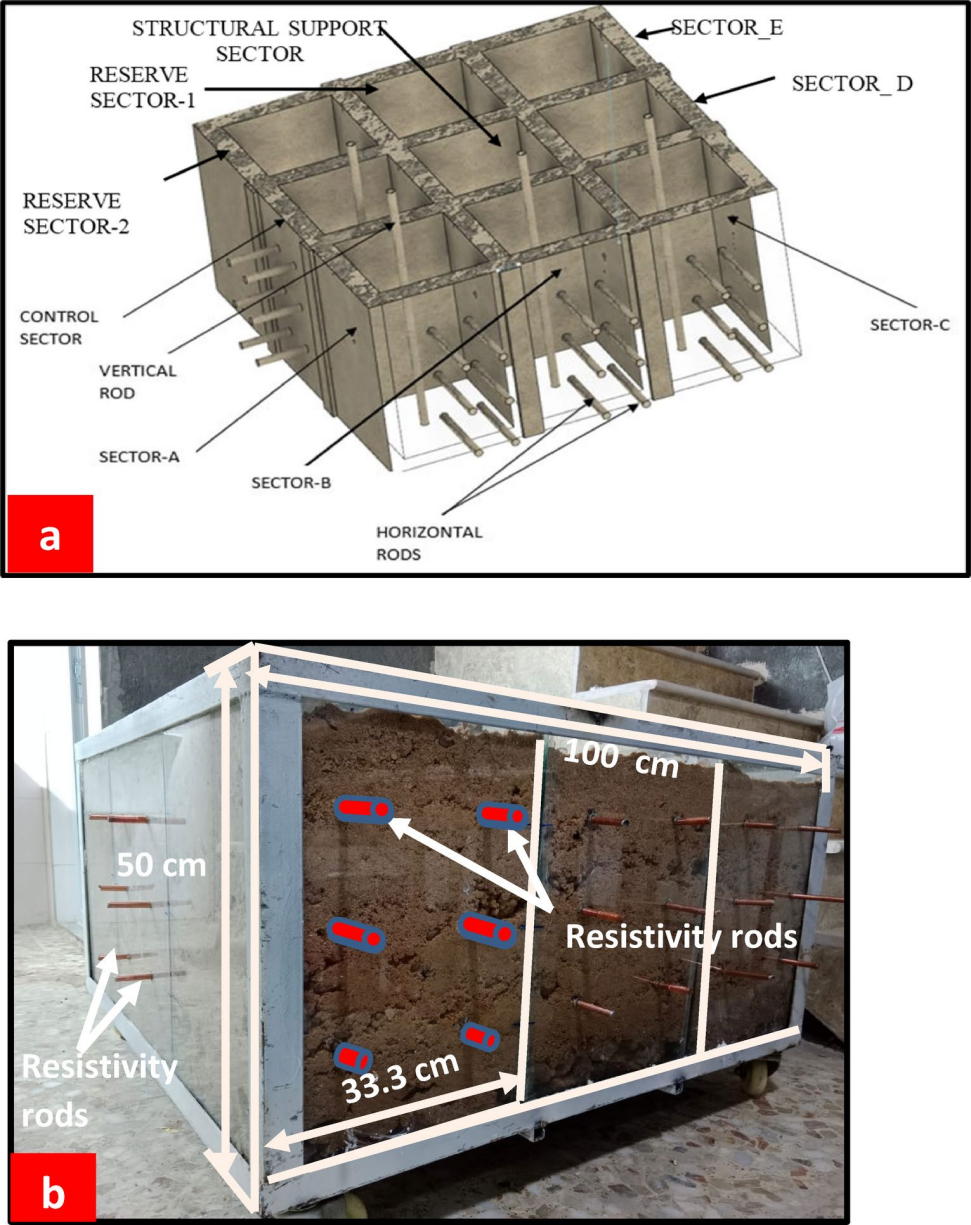
A glass soil box with a metal frame: (**a**) the schematic representation showing the division into sectors-scheme was generated with **Autodesk Fusion 360**, https://www.autodesk.com/products/fusion-360 (**b**) a photograph of the actual soil box, filled with stratified soil layers to conduct multiple LNAPL-related experiments.

Experiments were conducted within a glass soil box constructed with 1 cm-thick glass, featuring internal dimensions of 100 cm (length) × 100 cm (width) × 50 cm (height) as in Fig. 2. The soil box was equally divided into 9 sectors, each sector is 33.3 cm (length)×33.3 cm (width) x 50 cm (height). Two active sides of the box were selected for this experiment, one containing sectors A, B, and C, utilized for LNAPL spillage at varying flow rates of 5 ml/min, 25 ml/min, and 50 ml/min respectively. The other active side accommodated only one sector, functioning as a control sector. The rest five sectors were planned for advanced LNAPL experiments.

### Horizontal and vertical rods placement

**Fig. 3:**
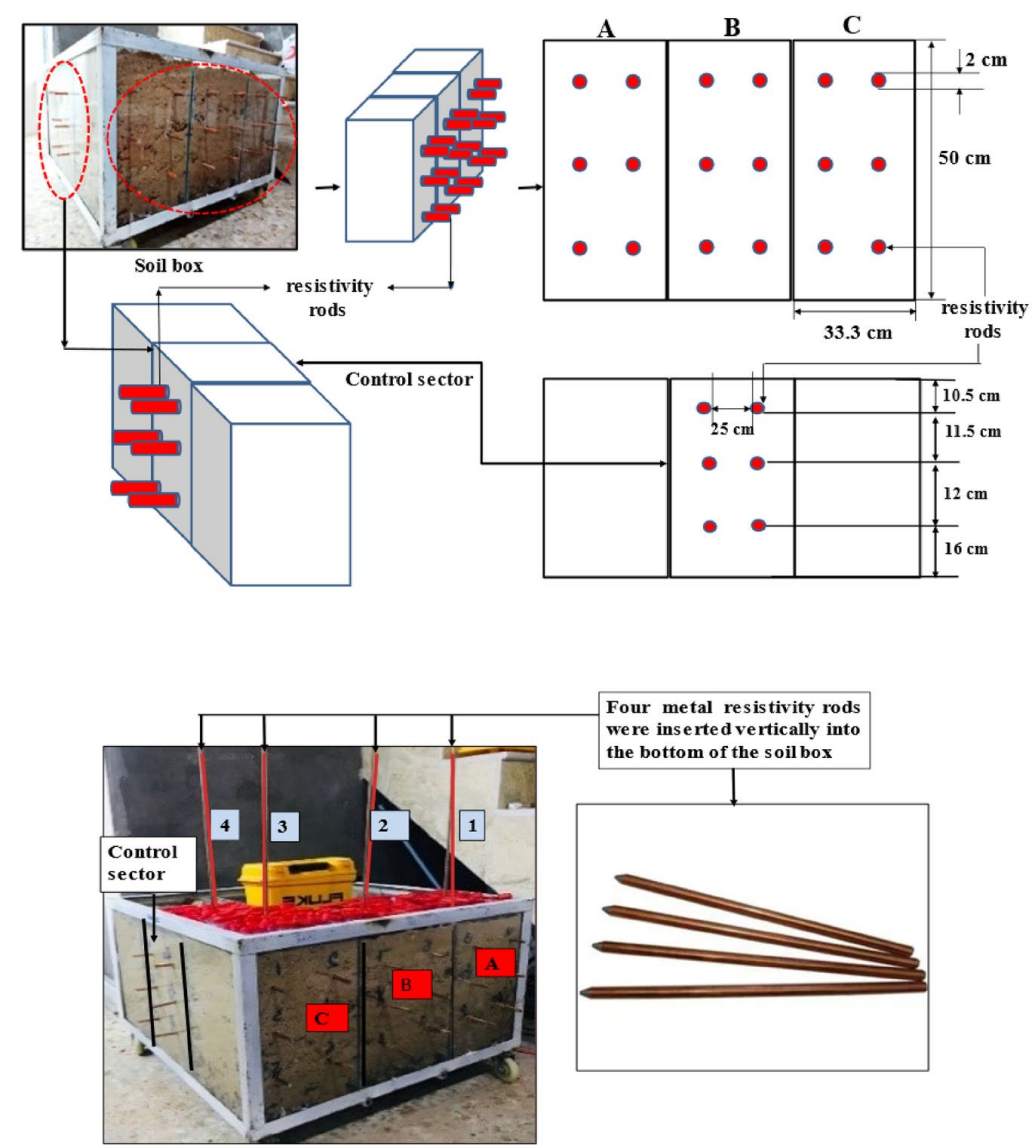
Horizontal and Vertical placement of Resistivity rods.

Twenty-four metal current-voltage rods were positioned on the outer sides of the box, eighteen rods were placed on the LNAPL injection side inserting them into the 2 cm holes, made on glass, the distance between each two rods was 25 cm for every two rods positioned horizontally in each sector, with six rods allocated to each sector, and six additional rods on the control sector’s side as illustrated in Fig. 3. Additionally, four reference rods were vertically installed on the box’s top side, aligned with the midpoint of each horizontal rod, and firmly embedded into the sand until reaching the box’s base.

Considering how soil composition, moisture content, and temperature affect soil resistivity, it’s important to note that moisture levels change with the seasons and vary at different depths in the soil. Additionally, temperature and water tend to remain more consistent at deeper layers. Based on findings by^32,33^ They suggested placing ground rods (or reference rods) as deeply as possible.

#### Soil layering

Soil samples (group 2) were packed in the glass soil box, and each layer’s volume amounted to 0.018 m^3^ upon packing the soil in the soil box, A four-layer pattern mirroring the original simple soil stratification at the field site was employed. The layers (L1, L2, L3, and L4) were designed with a thickness reduction factor of 1:3 to mimic the natural soil formation. L4 specifically represents the groundwater aquifer, permitting the movement of LNAPL.

We kept the soil moist enough before we began the experiment since it affects its electrical resistivity^34–36^ Therefore, it was covered loosely with a thin plastic sheet to retain moisture and allow for soil aeration.

### Soil profile assessment

To gain an overall understanding of the geotechnical properties of soil, a soil profile assessment was conducted. Group 1 soil samples were subjected to three important tests as follows:

- Time-sensitive tests such as pH, Relative Humidity RH%, Electrical Conductivity (EC), and hydraulic conductivity (k) tests were conducted according to^37–40^ to minimize potential alterations in soil properties and ensure the reliability of experimental results, test results are listed in Table 1.

**Table 1:** Time-sensitive soil testing.

Layers	RH%	PH	EC (MS/cm)	k (cm/sec)
L1	33	7.84	2.04	1.013
L2	35	7.83	1.841	1.074
L3	96	8.16	2.9	1.651
L4	72	8.19	3.2	1.869

The data from the Table 1 shows varying soil properties across four layers. Moisture content and the amount of dissolved minerals or contaminants increase in the deeper layers (L3 and L4). In contrast, the upper layers (L1 and L2) have lower moisture and fewer dissolved substances, which may influence how these layers interact with and affect the movement of contaminants like LNAPL. Understanding these variations is crucial for effectively managing environmental impacts, particularly in strategies designed to monitor and remediate areas affected by LNAPL spillages.

b. soil composition test was made according to^41^ Unified Soil Classification System (USCS) detailed information regarding soil texture and layer characteristics is illustrated in Table 2.

**Table 2:** Soil composition.

Layers	Type	Sand (%)	Silt (%)	Clay (%)
L1	loam	49.2	50	0.8
L2	Sandy loam	50	50	0
L3	loam	49.2	50	0.8
L4	Sandy loam	50	50	0

Table 2 outlines the composition of four soil layers identified as loam and sandy loam. Both loam layers (L1 and L3) contain 49.2% sand and 50% silt with a minimal clay content of 0.8%, suggesting moderate permeability due to a balanced mixture of particle sizes. The sandy loam layers (L2 and L4) have an equal proportion of sand and silt at 50% each but contain no clay, which likely enhances their permeability compared to the loam layers. This soil composition impacts how fluids, like LNAPL, infiltrate and move through the soil, with sandy loam allowing slightly faster movement than loam due to fewer fine particles and more considerable pore spaces.

c. soil properties tests were conducted to evaluate the effect of soil properties on the transport of Light Non-Aqueous Phase Liquids (LNAPLs) in the subsurface^42^, as presented in Table 3.

The table shows key soil properties for four layers, which are important for understanding how LNAPL moves underground. The **porosity** of the soil ranges from 59 to 60.4%, which means these soils have a lot of space for LNAPL to move through easily. **Particle density** varies slightly from 2.585 to 2.756 g/ cm^3^, affecting how tightly packed the soil particles are; higher values slightly slow down LNAPL movement.

**Bulk density** is between 1.047 and 1.114 g/cm³, indicating that the soil isn’t very compact. Softer, less dense soils allow LNAPL to spread faster and farther. The **Total Organic Carbon (TOC)** is extremely low (0.000025–0.00005%), which means there’s hardly any organic material in the soil to help break down the LNAPL naturally.

In summary, these soils are generally good at letting LNAPL flow through them because they are porous and not very dense^7,43,44^ All test was performed according to^45–48^ .

**Table 3:** Physical and chemical properties.

Soil layers	Porosity (%)	Particle density (g/ cm^3^)	Bulk density (g/cm^3^)	TOC (wt%)
L1	59.5	2.585	1.047	0.00005
L2	59	2.675	1.097	0.00005
L3	60.4	2.668	1.058	0.000025
L4	59.6	2.756	1.114	0.000031

### Electrical testing setup

**Fig. 4:**
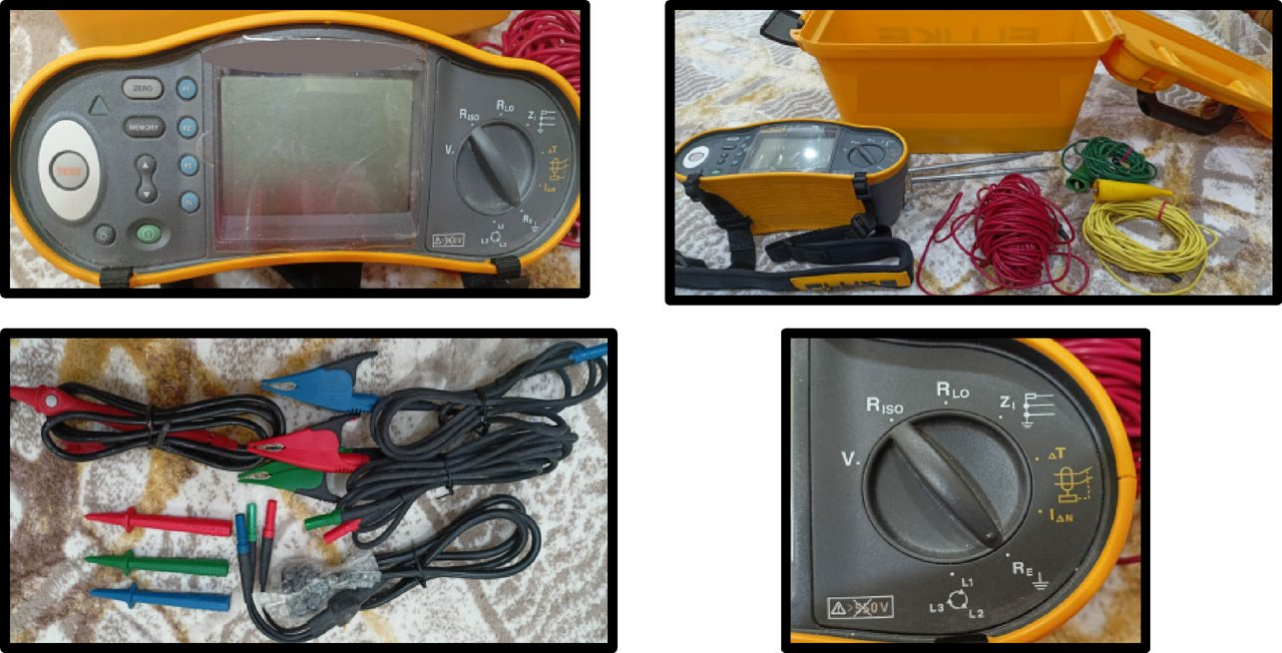
Fluke 1652 C Multifunction Installation Tester (USA).

The experimental sandbox used a Fluke 1652 C Multifunction Installation Tester (shown in Fig. 4), which is ideal for Electrical Resistivity (ER) testing. This device is known for its precise measurements and ease of use. It reliably provides a steady electric current at specific frequencies for each soil layer by connecting to pairs of rods. To ensure accurate readings without interference from ground currents, the tester features an Automatic Frequency Control (AFC) System. This system automatically chooses the best frequency that minimizes noise, enhancing the clarity and accuracy of the results^21,49,50^.

The measuring procedure used the universally accepted Wenner method developed by Dr. Frank Wenner of the US Bureau of Standards in 1915^13,51,52^ Which is one of the methods used to acquire soil resistivity measurements. This method offers stability in electrical resistivity measurements for soil studies, it is also called the four-pin method or four-probe method. It can be described as injecting a known current into the soil and measuring a voltage.

#### Data calculations

The electrical resistivity is calculated using the Wenner formula:

1 $${\uprho } = 2{\uppi } \times {\text{L}} \times {\text{R}}$$

Where ρ is the electrical resistivity in ohm-centimeters, L is the distance between two rods in centimeters, and R is the resistance in ohms.

#### Use of diesel as LNAPL surrogate

Diesel was chosen as a surrogate for LNAPL in this study due to its similar behavior and properties. Humidity and temperature were controlled throughout the experiments to ensure the accuracy of the results. This approach allowed for the accurate simulation of LNAPL migration without the complexities associated with using real contaminants. Details on the diesel properties and supporting studies^16,22,53^. The properties of fuel were tested according to^54^ and illustrated in Table 4.

**Table 4:** Diesel properties.

Fuel type	Fuel use	Density (g/cm3)	Viscosity (cP)	Boiling point range (°C)	Interfacial tension (mN/m)
Diesel	Transport fuel	0.87	2.7	150–370	50

#### Temporal analysis and monitoring of LNAPL introduction

After the delicate and complete packing of (group 2) soil samples, the Diesel was injected into the three active sectors at different flow rates 5 ml/min, 25 ml/min, and 50 ml/min., by injection syringe 20 ml with a needle gauge (diameter) between 18 and 22 gauge.

The tester (Fluke 1652 C) was connected to each of the two rods in one layer at a time for all active sectors. The tester was producing an electric current and receiving a voltage signal with a time interval of 4 h for a 24-hour duration. At the same time, 126 soil samples of approximately. (12–20) grams in quantity were methodically collected from two specific points within the central regions of each layer’s rectangular plane across all sectors. Utilizing a specialized spatula to ensure an accurate and representative sample for further analysis, samples were analyzed for LNAPL (diesel) concentrations according to^55,56^.

### Statistical analysis

IBM SPSS Statistics (version 29.0.2.0 (2020)) was used for data analysis.

Normality tests were used to assess data distribution, and correlation analysis was conducted to examine the relationships between variables.

Three distinct types of non-linear regression models were employed to investigate the relationships between ER values and LNAPL concentration.

The significance values were set to be less than 0.05, and a time series analysis was created to clarify the trends regarding the observations of ER (Electrical Resistivity) values.

## Results and discussion

This section presents all the study findings regarding the impact of LNAPL (Light Non-Aqueous Phase Liquid) release on electrical resistivity (ER) values in subsurface soil. The investigation focused on assessing the changes in ER values over time affected by LNAPL addition and examining the relationship between LNAPL concentration and ER values.

First, an overview of the ER values observed in the control sector will be presented and interpreted, followed by a detailed analysis of the temporal changes in ER values across all sectors. Additionally, more details are focused on Sector Cand B, where 50 ml and 25 ml of LNAPL were introduced, as it yielded notable results in the analysis. Furthermore, this study explored the application of non-linear regression models to fit the observed data on LNAPL concentration^57^and Electrical Resistivity (ER) values. And to determine the strength and the direction of the relationship.

### Control sector analysis

After analyzing the electrical resistivity data collected over 24 h, it was found that the soil in the control sector remained consistent in its resistivity levels. This suggests that moisture was evenly distributed and the soil was uniform throughout any minor changes that were observed, settled within the first 12 h^44,58^.

It was also noticed that the top layer consistently had lower resistivity than the deeper layers, which indicates differences in soil composition or moisture content, as shown in Table 5.

Overall, the stable resistivity values reassured that the soil was clean and the data collection was reliable, which provided a solid starting point for future geophysical investigations^59^.

**Table 5:** Resistivity values for the control sector in ohm-cm.

Time hrs.	ER-L1 Ω.cm	ER-L2 Ω.cm	ER-L3 Ω.cm
0	15,700	21,100	33,300
4	15,700	21,100	33,000
8	15,700	21,100	33,100
12	15,800	21,100	33,200
16	15,800	21,400	31,500
20	15,800	21,500	33,100
24	15,900	21,700	31,500
32	15,900	21,700	31,500

### Soil box layers analysis

Following the establishment of a stable baseline with the control sector, attention is directed towards the contaminated sectors. The analysis is structured to systematically examine each layer, starting with the first layer in sectors A, B, and C, followed by the second and third layers. This method helps track how soil resistivity changes at various depths because of LNAPL contamination.

#### Layer (L1)

**Fig. 5:**
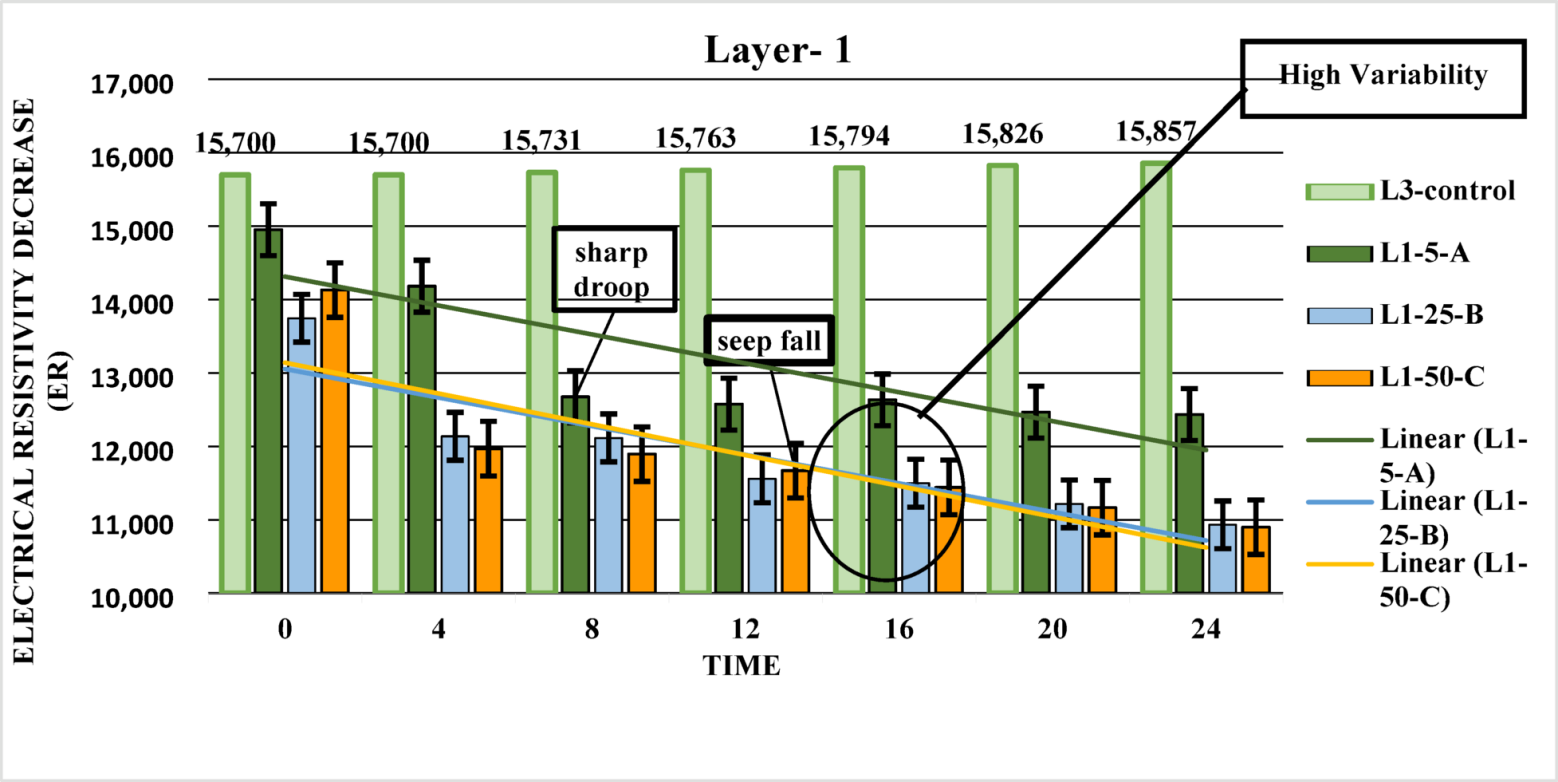
Electrical Resistivity Decrease Over Time in Layer-1: A Comparison of Different LNAPL Concentrations.

The graph illustrates two key trends in electrical resistivity in Layer 1: a ‘sharp drop,’ which is a rapid and large decrease around the 12-hour mark, indicating a sudden influx of LNAPL, followed by a ‘seep fall,’ a slower, steady decrease that indicates LNAPL continues to move through the soil. These observations align with the high hydraulic conductivity and sandy composition of Layer L1. Compared to Layers L3 and L4, Layer L1’s higher hydraulic conductivity and moderate electrical conductivity (EC) value suggest that LNAPL can move more readily in this layer. Additionally, the slightly acidic pH of Layer L1 has a minimal impact on LNAPL behavior and does not significantly hinder its movement. Overall, from the initial to the final time point (0 h to 24 h), there is a noticeable decrease in electrical resistivity across all sectors, demonstrating the spread of LNAPL within the layer (Fig. 5).

#### Layer( L2)

**Fig. 6:**
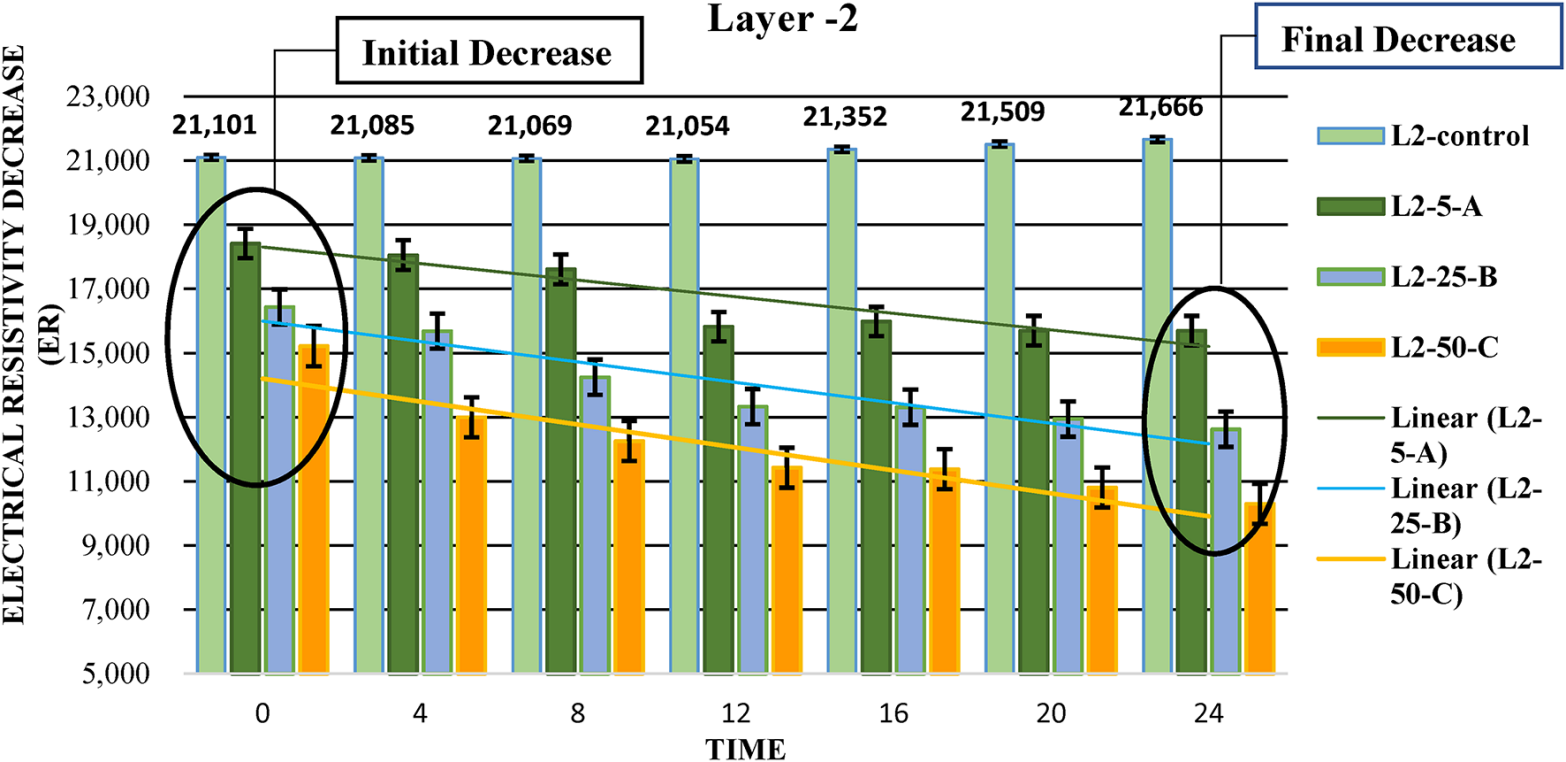
Electrical resistivity decrease over time in Layer-2: initial and final decrease trends.

As electrical resistivity decreases significantly in Layer 1 with higher LNAPL concentrations, a similar trend is seen in Layer 2 for all three sectors, as shown in Fig. 6, where L2-5-A, L2-5-B, and L2-50-C, experience noticeable drops in resistivity over time.

The chart shows a more consistent and predictable response, which could indicate a more uniform soil composition or a different interaction with the contaminants, possibly due to differences in soil texture, porosity, or chemical properties.

The “Initial Decrease” at the 0-hour mark shows the immediate impact of LNAPL on soil resistivity, indicating how quickly the soil’s electrical properties change upon exposure. The “Final Decrease” at the 24-hour mark reflects the long-term effects of LNAPL, providing a measure of the overall resistivity reduction after a day. These points illustrate the soil resistivity dynamics in response to LNAPL over time.

The lower hydraulic conductivity and sandy soil composition of Layer L2 compared to L1 may result in slower LNAPL movement. However, the moderate EC value still indicates some permeability, supporting potential LNAPL migration. The slightly acidic pH is again unlikely to have a significant effect on LNAPL behavior.

#### Layer (L3)

**Fig. 7:**
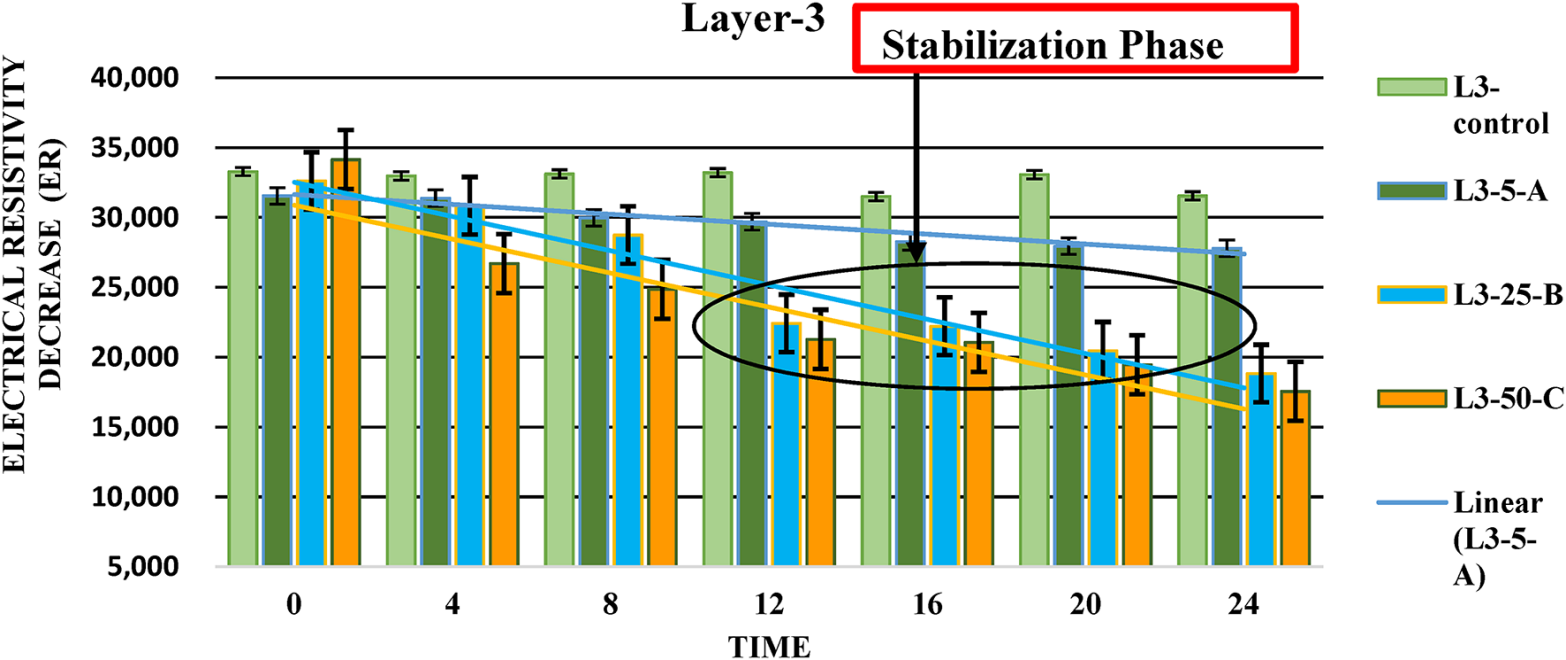
Electrical resistivity decrease over time in Layer-3: identification of the stabilization phases.

Observations from Layer 3 present a clear pattern in Fig. 7: as the amount of LNAPL increases, the soil resistivity decreases sharply over 24 h, compared to the first two layers.

Subsequent Sharp Decrease: As the experiment progresses, the sharp drop in resistivity for L3-5-A, becoming the lowest among the setups, suggests a rapid and significant interaction between the LNAPL and the soil. This could be due to the LNAPL dispersing more thoroughly within this setup, overcoming initial resistance and leading to greater conductivity. It’s also possible that the soil in L3-5-A has properties that facilitate faster or more extensive spread of LNAPL, such as higher permeability or lower retention capacity^60^, which could accelerate the decrease in resistivity.

Another important point that needs to be highlighted is that electrical resistivity values tend to stabilize or fluctuate slightly over time, especially in sectors C and B which may have higher permeability due to the sand-dominated composition and higher EC value than L1 and L2. The slightly alkaline pH may have minimal impact on LNAPL movement but could potentially facilitate its migration similar to the results found by^8,61^ .

As the electrical resistivity decreases consistently, it suggests that LNAPL may be seeping into the soil layers, similar to the findings of^24^.

The penetration depth of LNAPL in the saturated zone is affected by the volume of LNAPL released into the subsurface so an increase in the volume of LNAPL will increase the depth of penetration according to^35^above capillary fringe. this is why we used in this study two LNAPL volumes 25 ml and 50 ml to compare how fast the migration would be if an LNAPL was spilled at the same time in double amount^62^.

### Error bar analysis across layers

The error bars shown in Figures, 4, 5 and 6 represent the standard deviation (± SD) based on three replicates (*n* = 3), reflecting the variability between measurements and the consistency of the data. In **Layer 1**, error bars were relatively short at the initial stages, indicating consistent electrical resistivity values across replicates. However, the error bars gradually increased over time, particularly in L1-5-A and L1-25-B, suggesting greater variability due to uneven LNAPL migration and localized saturation effects. In **Layer 2**, error bars were short during the initial phase but became noticeably larger towards the final stages, especially in L2-5-A, indicating increased heterogeneity in LNAPL distribution as it migrated deeper. In **Layer 3**, the error bars were generally shorter than the upper layers, particularly after 12 h, indicating improved measurement consistency. This suggests that LNAPL migration became more stable at greater depths, with less surface disturbance or external influence. Overall, the error bars across all layers remained within acceptable scientific limits, supporting the reliability of the collected data.

### Assessing the relationship between ER values and LNAPL concentration

#### Experimental results

In this section, the numerical results of ER values and LNAPL concentration obtained from the temporal experiment are presented with their standard deviation for sectors B and C in Tables 6 and 7 as they illustrate a descriptive statistic for six bivariate relationships for six layers, followed by regression model analysis results, so it is important to clear that 84 out of 126 soil samples responded to chemical analysis to where a 50 ml and 25 ml LNAPL release was introduced.

**Table 6:** Descriptive statistics _sector C.

	L1_C		L2_C			L3_C		
Time hrs.	ER mean ± SD Ω.cm	LNAPL ± SD µg/g	ER mean ± SD Ω.cm		LNAPL ± SD µg/g	ER mean ± SD Ω.cm		LNAPL ± SD µg/g
0	14128.7 ± 1055.59	13.1 ± 1.27	15218.5 ± 1095.4		20.1 ± 0.61	34143.21 ± 3544.5		37.6 ± 11.02
4	11968.7 ± 162.9	13.21 ± 2.7	12999.4 ± 544.16		30.02 ± 5.50	26696.7 ± 1527.4		38.4 ± 4.23
8	11893.9 ± 456.9	17.69 ± 3.13	12256.7 ± 616.6		33.2 ± 0.02	24858.05 ± 1892.1		41.6 ± 0.67
12	11670.1 ± 333.3	19.9 ± 2.11	11426.6 ± 656.1		30.29 ± 6.54	21283.7 ± 2312.8		42.14 ± 14.19
16	11440.5 ± 333.35	19.81 ± 1.67	11376.1 ± 377.31		36.21 ± 1.27	21060.3 ± 1536.9		42.89 ± 3.435
20	11164.8 ± 119.1	20.74 ± 1.69	10805.6 ± 195.39		37.57 ± 0.55	19462.9 ± 441.15		42.7 ± 0.45
24	10898.4 ± 106.53	20.6 ± 1.78	10298.81 ± 78.30		37.35 ± 3.25	17560.7 ± 225.9		46.97 ± 6.24

**Table 7:** Descriptive statistics_ sector B.

	L1_B		L2_B		L3_B	
Time hrs.	ER mean ± SD Ω.cm	LNAPL ± SD µg/g	ER mean ± SD Ω .cm	LNAPL ± SD µg/g	ER mean ± SD Ω.cm	LNAPL ± SD µg/g
0	13745.2 ± 749.45	48.25 ± 21.75	158.1 ± 1.42	17.5 ± 2.5	16437.8 ± 455.3	17.1 ± 2.89
4	12137.2 ± 121.4	55.5 ± 20.5	158.42 ± 5.09	40 ± 30	15685.1 ± 887.6	20.5 ± 3.66
8	12114.9 ± 303.6	57.5 ± 22.5	143.53 ± 3.83	45 ± 2	14248.3 ± 570.7	20.4 ± 0.371
12	11558.4 ± 358.27	59.5 ± 22.5	128.6 ± 5.72	51.5 ± 1.5	13331.1 ± 687.9	20.35 ± 1.067
16	11497.97 ± 244.5	59.25 ± 22.75	125.8 ± 5.33	61 ± 1	13312.1 ± 531.82	24.6 ± 5.584
20	11216.13 ± 97.07	62.5 ± 22.5	126.32 ± 1.22	58.5 ± 5.5	12945.9 ± 191.50	28.023 ± 0.98
24	10931.7 ± 46.96	69.5 ± 20.5	129.4 ± 1.6	42.5 ± 27.5	12624.5 ± 212.6	28.5 ± 4.54

#### Exploratory data analysis

To ensure the reliability of our regression models, preliminary analyses were conducted, including normality tests and correlation assessments. The normality tests confirmed that the residuals met the assumptions required for valid regression analysis, indicating that the error terms were normally distributed. Additionally, correlation analysis was performed to check for multicollinearity among independent variables, ensuring that each variable uniquely contributed to the model.

In this study, electrical resistivity (ER) was considered the independent variable, while LNAPL concentration was treated as the dependent variable. This classification is justified by the fundamental principle that ER changes in response to subsurface conditions, including fluid content and composition. Since LNAPL intrusion alters the soil’s resistivity, ER serves as a predictive factor influencing the observed LNAPL concentration. By structuring the model this way, we ensured that ER variations could be used to infer LNAPL behavior, reinforcing the reliability of our regression models and supporting the validity of our findings.

#### Selection of regression models

Many environmental studies^57,63,64^ showed that Linear models often fail to capture the complex realities of environmental contamination, as changes in subsurface soil and contaminant migration are not always straightforward. Real-world data frequently show significant deviations from these models. Thus, incorporating nonlinear relationships into spill management can improve geotechnical planning similar findings by^65^ suggested that considering nonlinear relationships in natural incidents spillage management can help in planning nonlinear geotechnical dynamics.

In line with these findings, simple to advanced regression models were employed to match the complex patterns observed in the interaction between electrical resistivity (ER) and LNAPL concentration data. Quadratic, logarithmic, and power models were selected based on their capability to best represent these observed interactions. Each model was chosen to accurately reflect the varying impacts of LNAPL concentration on ER, ensuring that the analysis was thorough and precise.

#### Nonlinear regression analysis

Before interpreting the nonlinear regression analysis, it is important to clarify that this study employs the statistical parameters presented in Table 8 to evaluate the model’s fitness.

**Table 8:** Study statistical parameters.

Parameter	Description
F-value	Assesses the overall significance of the regression model. A higher F-value indicates that the model effectively explains the variation in LNAPL concentration
R^2^ (Coefficient of Determination)	Represents the proportion of variance in LNAPL concentration that is explained by ER. A higher R² (closer to 1) suggests a stronger correlation.
P-value	Determines the statistical significance of the regression model. A p-value below 0.05 indicates that the relationship between ER and LNAPL concentration is statistically significant
F	Tests the overall significance of the regression model. A higher F-value indicates a better model fit
b1 (Primary Coefficient)	Represents the direct effect of ER on LNAPL concentration. In linear regression models, it defines the rate of change in LNAPL concentration per unit change in ER
b2 (Quadratic Coefficient)	Applies to quadratic models, capturing curvature in the relationship. A significant b2 suggests a nonlinear trend in LNAPL migration
b3 (Cubic Coefficient)	Used in cubic models to describe more complex relationships. Not applicable in this study

#### Sector B statistical analysis

This section presents the statistical analysis of Sector B’s stratified layers, progressing from the surface (L1) to the deepest layer (L3), As shown in Table 9: For Layer 1 (L1), the quadratic model had the (highest R² value 0.942, *p* = 0.003, F = 32.579), suggesting that ER decreases with increasing LNAPL concentration in a nonlinear trend, with an initial rapid drop followed by a stabilization phase. The power model (R^2^ = 0.925, *p* < 0.001, F = 61.842) also showed strong predictive capability, reinforcing that LNAPL migration near the surface follows a nonlinear resistivity reduction pattern. The logarithmic model, with significant (R^2^ = 0.89, *p* = 0.001, F = 40.397), was slightly weaker in explaining the variations in LNAPL concentration.

**Table 9:** Regression models parameters _sector _B.

Sector	Layer	Model	F	*R* ^2^	*P*-value	b1	b2	b3
B	L1	Logarithmic	40.397	0.89	0.001	-81.182	N/A	N/A
		Quadratic	32.579	0.942	0.003	-0.059	2.10E-06	N/A
		Power	61.842	0.925	< 0.001	-1.423	N/A	N/A
	L2	Logarithmic	4.731	0.486	0.082	-108.398	N/A	N/A
		Quadratic	12.047	0.858	0.02	0.12	-4.68E-06	N/A
		Power	6.329	0.559	0.053	-3.366	N/A	N/A
	L3	Logarithmic	8.692	0.685	0.042	-16.189	N/A	N/A
		Quadratic	3.45	0.697	0.167	-0.003	5.02E-08	N/A
		Power	9.189	0.697	0.039	-0.73	N/A	N/A

In Layer 2 (L2), the quadratic model again provided the best fit (R^2^ = 0.858, *p* = 0.02, F = 12.047), indicating a slower migration rate due to soil heterogeneity and retention effects. The logarithmic model (R^2^ = 0.486, *p* = 0.082, F = 4.731) and power model (R^2^ = 0.559, *p* = 0.053, F = 6.329) were less predictive, suggesting higher variability in LNAPL behavior within this layer.

For Layer 3 (L3), the power model was the most suitable (R^2^ = 0.697, *p* = 0.039, F = 9.189), indicating a stabilization phase in LNAPL migration as it reaches the water table, causing ER values to level off. The logarithmic model (R^2^ = 0.685, *p* = 0.042, F = 8.692) performed similarly, suggesting some degree of fit, while the quadratic model (R^2^ = 0.697, *p* = 0.167, F = 3.45) was not statistically significant, indicating that a quadratic trend does not accurately capture LNAPL behavior in this deeper layer.

The negative b1 values confirm a strong inverse relationship between ER and LNAPL concentration, indicating that as LNAPL accumulates, resistivity decreases. The correlation is strongest in L1, where LNAPL migrates freely, while L2 shows a weaker but significant trend due to retention effects. In L3, the relationship stabilizes, suggesting limited further migration due to capillary forces and permeability barriers.

Finally, while the quadratic model worked well for L1 and L2, the power model is more versatile and physically realistic, making it the preferred choice for understanding LNAPL migration trends in subsurface environments.

#### Sector C statistical analysis

Having established the nonlinear regression trends in Sector B, the analysis now shifts to Sector C, where the LNAPL spill volume was doubled. This section examines whether increased spill volume influences the resistivity-LNAPL relationship and alters migration behavior.

Table 10 shows that In Layer 1 (L1), the logarithmic model (R^2^ = 0.655, *p* = 0.027, F = 9.495) and power model (R^2^ = 0.652, *p* = 0.028, F = 9.36) showed the strongest performance, suggesting a rapid initial ER decrease followed by stabilization. The quadratic model (R^2^ = 0.723, *p* = 0.076, F = 5.231) was not statistically significant, indicating it does not fully capture LNAPL behavior in this layer.

**Table 10:** Regression models parameters _sector _C.

Sector	Layer	Model	F	*R* ^2^	*P*-value	b1	b2	b3
C	L1	Logarithmic	9.495	0.655	0.027	-32.12	N/A	N/A
		Quadratic	5.231	0.723	0.076	-0.028	1.01E-06	N/A
		Power	9.36	0.652	0.028	-1.944	N/A	N/A
	L2	Logarithmic	32.606	0.867	0.002	-43.767	N/A	N/A
		Quadratic	16.955	0.894	0.011	0.003	-2.70E-07	N/A
		Power	30.517	0.859	0.003	-1.551	N/A	N/A
	L3	Logarithmic	28.519	0.851	0.003	-12.895	N/A	N/A
		Quadratic	16.957	0.894	0.011	-0.002	3.35E-08	N/A
		Power	32.161	0.865	0.002	-0.311	N/A	N/A

For Layer 2 (L2), all models showed stronger fits compared to L1, with the logarithmic model (R^2^ = 0.867, *p* = 0.002, F = 32.606) and power model (R^2^ = 0.859, *p* = 0.003, F = 30.517) best describing the progressive resistivity decline. The quadratic model (R^2^ = 0.894, *p* = 0.011, F = 16.955) was significant but indicated possible variations in early migration.

In Layer 3 (L3), the power model (R^2^ = 0.865, *p* = 0.002, F = 32.161) best represented the stabilization phase where ER reduction slows due to lateral migration along the water table. The logarithmic model (R^2^ = 0.851, *p* = 0.003, F = 28.519) provided a similar trend, while the quadratic model (R^2^ = 0.894, *p* = 0.011, F = 16.957) was significant but less predictive.

The negative b1 values confirm a strong inverse relationship between ER and LNAPL concentration, meaning that as LNAPL accumulates, resistivity decreases. The relationship is moderate in L1, strongest in L2, and stabilizes in L3, reflecting the influence of permeability and capillary forces. Compared to Sector B, the larger LNAPL volume in Sector C intensified ER reduction, particularly in L2 and L3, reinforcing the role of spill volume in accelerating migration and resistivity changes.

The negative relationship between electrical resistivity (ER) and LNAPL concentration can be explained by soil properties affecting fluid conductivity. In L1 and L2 (loam & sandy loam), lower electrical conductivity (EC) (2.04–1.84 mS/cm) and moderate porosity (59–59.5%) result in higher initial ER values^66^. However, as LNAPL infiltrates, it displaces pore water, reducing ionic conduction and decreasing ER. In L3 and L4 (higher EC: 2.9–3.2 mS/cm, porosity: ~60%), the effect is more pronounced due to greater water retention and mineral content, which initially lowers ER but further declines as LNAPL saturation increases^67^. The presence of organic carbon (TOC), though minimal, also impacts charge mobility. Thus, soil composition, moisture content, and pore fluid conductivity collectively control ER behavior in response to LNAPL migration^68^.

The pH of the soil influences the ion exchange capacity and mobility of charged particles, which in turn affects electrical resistivity (ER). In this study, the pH ranges from 7.83 to 8.19, indicating slightly alkaline conditions.

Higher pH (L3 & L4: 8.16–8.19) tends to increase the availability of dissolved ions, which results in lower initial ER values. However, as LNAPL displaces pore water, the available ions decrease, leading to a further drop in ER.

Lower pH (L1 & L2: 7.83–7.84) correlates with lower EC, meaning these layers are less conductive initially. When LNAPL infiltrates, the reduction in water content further limits charge transport, causing a greater decrease in ER compared to higher-pH layers.

Thus, pH indirectly influences the ER-LNAPL relationship by regulating ionic mobility and fluid conductivity, with higher-pH layers showing a more pronounced ER drop due to greater initial ion availability.

In line with the findings of^22–25^ Our study observed a significant negative relationship between ER and LNAPL concentration across all layers, similarly^4,53,69–71^ identified that high resistivity values were consistently associated with the presence of LNAPL compared to non-LNAPL media and revealed a decrease in electrical resistivity measurements with an increase in LNAPL content. While the data shows clear trends, it is important to reflect on what these patterns mean in context. For example, some fluctuations in electrical resistivity or measurement accuracy may appear sudden or inconsistent at first. However, based on our hands-on observation during the experiments and our understanding of LNAPL behavior in unsaturated soils, we interpret these changes as the result of rapid redistribution, localized saturation effects, or interface interference between the contaminant and moisture within the soil.

Not all of these shifts can be backed by numerical validation from prior studies, as comparable datasets are often unavailable or not directly aligned with our approach. Still, this interpretation provides a logical and experience-based explanation for what we observed—and we believe it helps build a more complete understanding of the system’s behavior^72–74^.

## Conclusion

This research aims to develop a non-invasive method for detecting immediate LNAPL spillage through stratified soil layers and to establish the statistical relationship between electrical resistivity (ER) and LNAPL concentration. The conclusions drawn from the study are as follows:

- The ER-based detection method demonstrated high sensitivity to immediate LNAPL migration, making it an effective tool for real-time environmental monitoring.
- The statistical analysis confirmed a strong inverse relationship between ER and LNAPL concentration, with negative b1 (primary coefficient) values validating the impact of LNAPL accumulation on resistivity trends, particularly in intermediate soil layers, where the strongest correlation was observed.
- LNAPL spill volume significantly influenced resistivity trends. In the lower spill volume scenario, the quadratic model best described the upper and intermediate layers, while the power model captured the stabilization of ER in deeper layers near impermeable boundaries. In the higher spill volume scenario, resistivity reduction was more pronounced, particularly in deeper layers, with logarithmic and power models better explaining ER changes. These results confirm that larger spills accelerate migration and intensify resistivity changes.
- The power model was found to be the most reliable predictor across multiple soil depths, particularly in deeper layers where LNAPL movement slows due to permeability constraints. The quadratic model was effective in early-stage migration but less predictive at depth.
- The developed models are valid within the considered range of parameters, including b1 (primary coefficient), b2 (quadratic coefficient), and R² (coefficient of determination), but beyond this range, prediction accuracy should be verified to ensure model reliability under varying soil conditions.

These findings confirm that ER measurements can serve as a reliable, non-invasive tool for detecting and tracking LNAPL contamination.

- **Study limitation**.

While the exclusion of 42 samples from chemical analysis in Sector A is acknowledged as a limitation, it does not compromise the reliability of the study, as:

1. The ER-based detection method was consistently applied.
2. Findings from other sectors validate the observed trends.
3. The exclusion was necessary to maintain data integrity.
4. Statistical models remained robust despite the missing chemical data.

- **Future Studies and Recommendations.**
- Expanding Sample Size and Chemical Validation:
- Future research should incorporate a larger sample size and chemical validation across all sectors.
- Longer Monitoring Durations:
- Extending the observation period would allow for a more detailed analysis of long-term LNAPL behavior, including resistivity stabilization trends and delayed migration effects.
- Application of Alternative Statistical and ML Models:
- While this study utilized nonlinear regression, future studies could explore machine learning models (e.g., ANN, Random Forest) to enhance predictive accuracy while ensuring interpretability through feature importance analysis.
- Testing in Different Soil and Hydrogeological Conditions:
- To improve the applicability of the ER-based detection method, future research should assess its performance under varied soil types, moisture levels, and permeability conditions, ensuring adaptability to diverse environmental settings.
- Integration with Real-Time Monitoring Systems:
- The development of automated, real-time ER monitoring systems could provide continuous LNAPL detection, improving early warning capabilities in contamination-prone areas.

By addressing these aspects, future research can further enhance the accuracy, applicability, and predictive power of ER-based LNAPL detection methods in environmental monitoring and contamination assessment.

## Data Availability

Data availability statementThe data that support the findings of this study are available from the corresponding author upon reasonable request.
